# The TgAMPK-TgPFKII axis essentially regulates protein lactylation in the zoonotic parasite *Toxoplasma gondii*

**DOI:** 10.1128/spectrum.02044-24

**Published:** 2025-02-07

**Authors:** Chenghuan Li, Yang Zhao, Qilong Li, Ran Chen, Ying Feng, Xiaoyu Sang, Xiangrui Li, Bang Shen, Ning Jiang, Qijun Chen

**Affiliations:** 1 Key Laboratory of Livestock Infectious Diseases, Ministry of Education, and Key Laboratory of Ruminant Infectious Disease Prevention and Control (East), Ministry of Agriculture and Rural Affairs, College of Animal Science and Veterinary Medicine, Shenyang Agricultural University, Shenyang, China; 2 Research Unit for Pathogenic Mechanisms of Zoonotic Parasites, Chinese Academy of Medical Sciences, Shenyang, China; 3 College of Veterinary Medicine, Nanjing Agricultural University, Nanjing, Jiangsu, China; 4 State Key Laboratory of Agricultural Microbiology, College of Veterinary Medicine, Huazhong Agricultural University, Wuhan, Hubei, China; Clemson University, Clemson, South Carolina, USA

**Keywords:** *Toxoplasma gondii*, lactylation, TgPFKII, AMPK signaling pathway, AMPK inhibitor

## Abstract

*Toxoplasma gondii* infects nucleated cells of warm-blooded animals and cause zoonotic toxoplasmosis. Lysine lactylation, as a novel post-translational modification, is essential for epigenetic regulation and cellular processes, and proteomic analyses have shown that lactylated proteins are involved in a wide range of biological processes including energy metabolism, gene regulation, and protein biosynthesis. Additionally, protein lactylation is prevalent in *T. gondii*, while its regulatory mechanisms have not been fully understood. In this study, we investigated the role of *T. gondii* phosphofructokinase-2 (TgPFKII) and the adenosine-5’-monophosphate-activated protein kinase (AMPK) signaling pathway in the invasion, replication, and lactylation regulation of *T. gondii*. We localized TgPFKII in the cytoplasm of *T. gondii* tachyzoites and demonstrated its necessity for parasite growth and protein lactylation through auxin-induced degradation. Our results showed that inhibition of the AMPK pathway led to decreased TgPFKII expression and reduced protein lactylation levels. Furthermore, AMPK-specific inhibitors significantly impaired parasite invasion and proliferation. These findings highlight TgPFKII as a crucial regulator of lactylation and underscore the importance of the AMPK pathway in *T. gondii*’s pathogenic mechanisms, offering potential targets for therapeutic intervention.

**IMPORTANCE:**

Understanding the intricate mechanisms by which *Toxoplasma gondii* invades and proliferates within host cells is essential for developing novel therapeutic strategies against toxoplasmosis. This study focuses on the pivotal roles of *T. gondii* phosphofructokinase-2 (TgPFKII) and the adenosine-5’-monophosphate-activated protein kinase (AMPK) signaling pathway in regulating protein lactylation in association with parasite invasion and growth. By elucidating the cellular localization and functional importance of TgPFKII, as well as its regulation through AMPK-specific inhibitors, we provide comprehensive insights into the metabolic and signaling networks that underpin *T. gondii* pathogenicity. Our findings reveal that TgPFKII is a critical regulator of lactylation and that the AMPK pathway significantly influences *T. gondii*’s ability to invade and replicate within host cells. These insights pave the way for targeted interventions aimed at disrupting key metabolic and signaling pathways in *T. gondii*, potentially leading to more effective treatments for toxoplasmosis.

## INTRODUCTION

Lactate is a key energy source and glucose precursor in metabolism (1–3). Recent research indicates lactate can drive histone lactylation, regulating gene transcription beyond its metabolic role (4, 5). Lysine lactylation, a new protein post-translational modification, is crucial for epigenetic regulation and cellular processes (6–14). *Toxoplasma gondii*, a protozoan parasite infecting a third of the global population, has a complex carbon metabolism and a complete glycolytic pathway, enabling it to thrive in diverse host environments (15–17). Our and another previous study suggest that protein lactylation occurs widely in *T. gondii* RH strain, with 1,964 lactylation sites identified on 955 proteins (6, 18). These lactylated proteins are found in various cell compartments and participate in diverse biological processes, including energy metabolism, gene regulation, and protein biosynthesis (6, 18).

The mechanisms of lactylation modifications have been investigated in several studies (19, 20). It was found that overexpression of histone acetyltransferase (p300) in HEK293T cells led to increased levels of histone lactylation, and knockdown of p300 in HEK293T and HCT116 cells resulted in decreased levels of protein lactylation (11). Additionally, lactate-induced histone lactylation was significantly impaired upon p300 knockdown in mouse bone marrow-derived macrophages (21). These evidence suggest that p300, as a regulator of protein lactylation, can act as a “writer” of lactylation of specific proteins (4, 11, 19, 22). In contrast to the extensive research conducted on humans and model organisms, the regulation of lactylation modifications in *T. gondii* remain largely elusive.

Phosphofructokinase-2 (PFKII) is a key factor in coordinating catabolic and anabolic activities in *T. gondii* (6). Conditional knockdown of PFKII results in increased pyrophosphate (PPi) levels, but reduced macromolecular biosynthesis and glycolysis in *T. gondii* (6, 17). Our laboratory found that lactate-modified proteins were involved in energy metabolic processes such as glycolysis in *T. gondii*, and PFKII is the key enzyme in the glycolysis pathway and may be closely related to the lactylation of the parasite (6, 18). Thus, confirmation of the exact functions of PFKII in protein lactylation in *T. gondii*, especially its role as “writer” in this biological process, is important to understand the mechanisms of lactylation in *T. gondii*.

In mammals, adenosine-5’-monophosphate-activated protein kinase (AMPK), a heterotrimeric protein complex consisting of α, β, and γ subunits, is known as a highly conserved master regulator of metabolism (23, 24), and a sensor of cellular energy status (25, 26). The activation of AMPK is mainly dependent on the phosphorylation at a conserved threonine residue within the activation loop, and it functions also through phosphorylations of the target proteins. It has been reported that AMPK phosphorylates more than 100 distinct target proteins on at least 130 sites and modulates almost every branch of cell metabolisms (27), including lipid synthesis, oxidation, and lipolysis (24, 28). In eukaryotic cells, AMPK regulates glycolysis-related enzymes by autophosphorylation, including activation and phosphorylation of the glycolysis-related enzyme PFKII (27). Recently, it was reported that *T. gondii* AMPK was structurally similar to the mammal AMPK, which is also composed of α, β, and γ subunits (29). Conditional knockdown of TgAMPKα and TgAMPKγ reduced *T. gondii* motility, cell invasion capacity, and intracellular replication. Knockdown of TgAMPKβ impaired *T. gondii* cell cycle progression and parasite growth and development (29–31). It was also found that deletion of TgAMPKγ inhibited the phosphorylation of TgAMPKα, resulting in changes in the expression abundance of relevant metabolic enzymes for glycolysis and affecting the energy metabolism process in *T. gondii* (29). This suggests that *T. gondii* PFKII (TgPFKII) may also be regulated by the AMPK signaling pathway like eukaryotic cells. Therefore, studying the effects of AMPK signaling pathway on PFKII as well as on the protein lactylation in *T. gondii* is of great significance for the in-depth elucidation of the regulatory mechanism of PTM in *T. gondii*.

In this study, we show that deletion of the TgPFKII gene resulted in the reduction of general protein lactylation levels in *T. gondii*, suggesting that TgPFKII is an important regulator or “writer” for lactylation in the parasite. We also show that TgPFKII and protein lactylation were regulated by the AMPK pathway.

## RESULTS

### PFKII was localized in the cytoplasm of *T. gondii*

**Fig 1:**
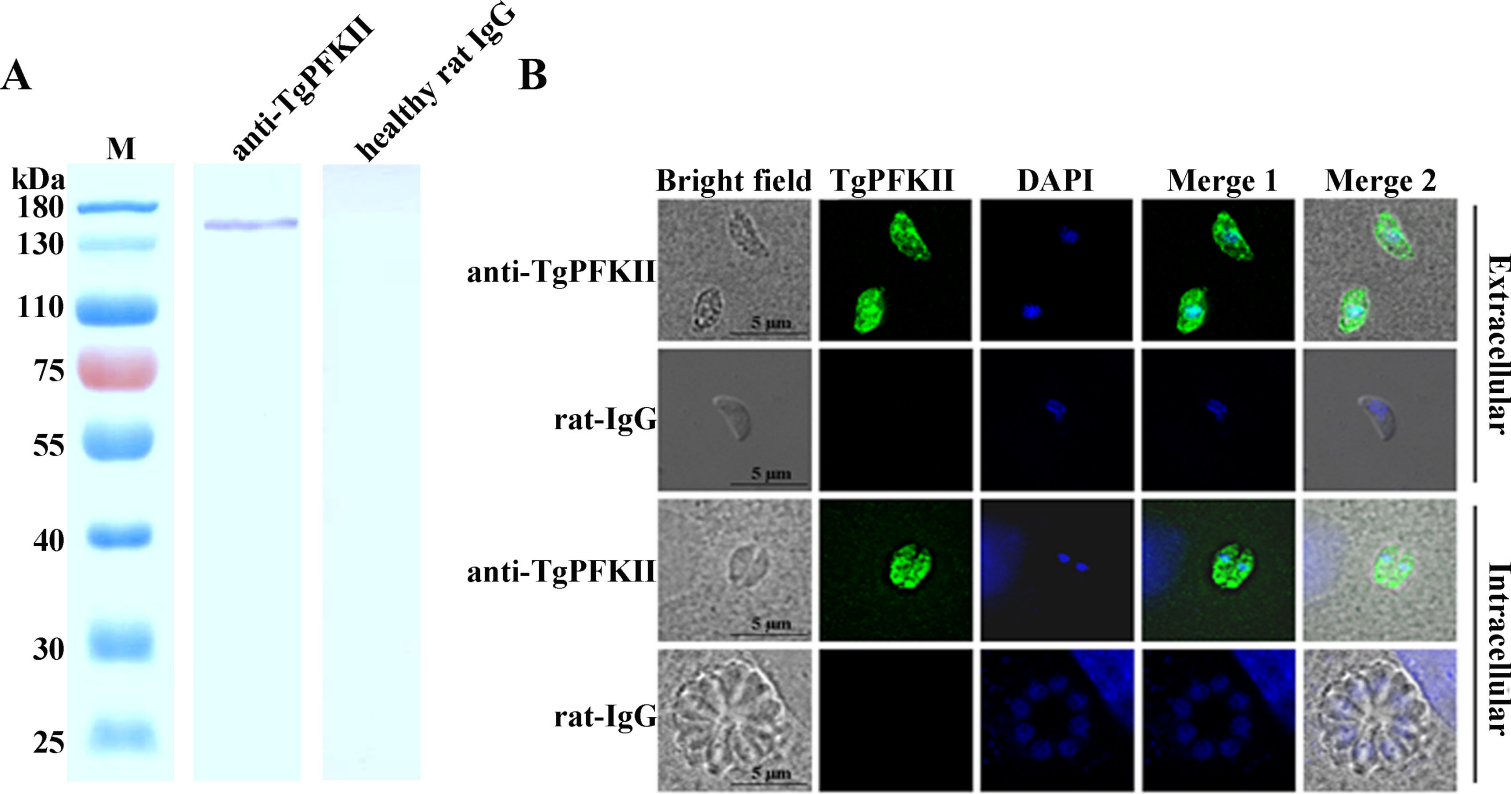
TgPFKII is localized in the cytoplasm of tachyzoites of *T. gondii*. (**A**) The expression of native PFKII in *T. gondii* RH-TIR1 strain was detected with PFKII-specific antibodies in Western blot assays. Sera of a healthy rat were used as the negative control. (**B**) The TgPFKII fluorescence signal (green) was localized in the cytoplasm of RH-TIR1 tachyzoites. Sera of a healthy rat were used as the negative control. Scale bar, 5 µm.

Determining the cellular localization of proteins is important for understanding their function. Therefore, to localize PFKII in *T. gondii*, we purified a His-tagged PFKII fusion protein (Fig. S1) and immunized rats to generate PFKII-specific polyclonal antibodies. We found that PFKII, approximately 131 kDa in *T. gondii* RH-TIR1 strain, was specifically detected by Western blotting using PFKII-specific antibody (Fig. 1A).

The localization of TgPFKII in *T. gondii* was further determined using an immunofluorescence assay (IFA). The fluorescent signal of TgPFKII was observed in the cytoplasm of *T. gondii* RH-TIR1 tachyzoites (Fig. 1B). The data demonstrated that TgPFKII was localized in the cytoplasm of *T. gondii*.

### TgPFKII is essential for the growth of *T. gondii*

**Fig 2:**
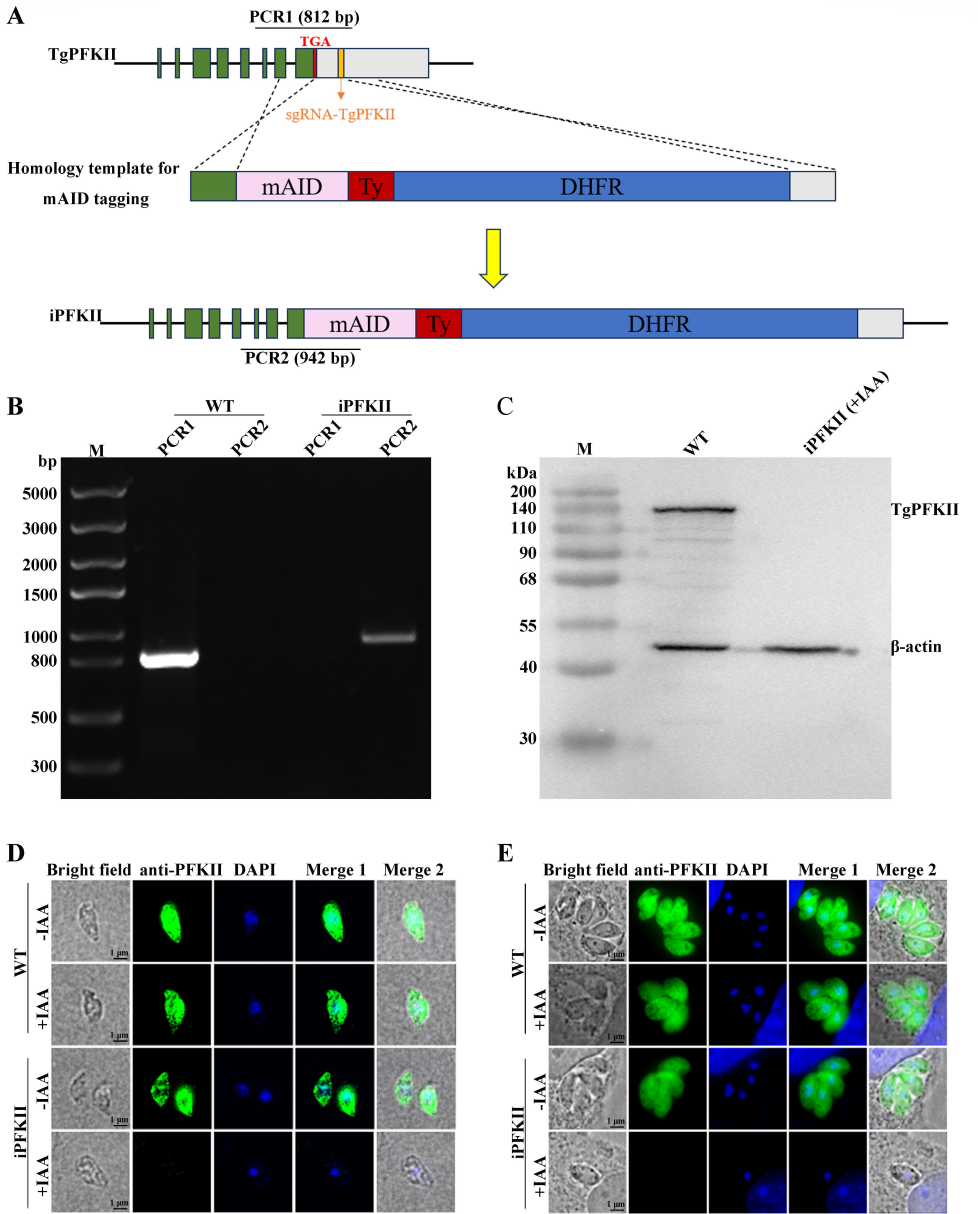
Construction of a PFKII depletion strain. (**A**) PFKII was fused with a mAID tag at the C-terminus of RH-TIR1 strain. Yellow bar indicates CRISPR targeting sites. (**B**) The monoclonal strain iPFKII was confirmed by PCR. WT was the RH-TIR1 strain. (**C**) The degradation of PFKII after IAA treatment of iPFKII was examined by Western blotting. β-Actin was used for normalization. (**D and E**) PFKII expression was examined by indirect immunofluorescence in intracellular and extracellular parasites. Scale bar, 1 µm.

To further investigate the role of TgPFKII, we employed auxin-induced degradation (AID) to remove TgPFKII from the parasite. A mini-AID tag (mAID) was added to the C-terminus of the PFKII (Fig. 2A). And the monoclonal strain iPFKII was identified by using polymerase chain reaction (PCR) (Fig. 2B). After 4 hours of indole-3-acetic acid (IAA) treatment, PFKII was undetectable by Western blotting using an anti-PFKII antibody (Fig. 2C). Immunofluorescence also corroborated the above results (Fig. 2D and E, Fig. S5A and B).

**Fig 3:**
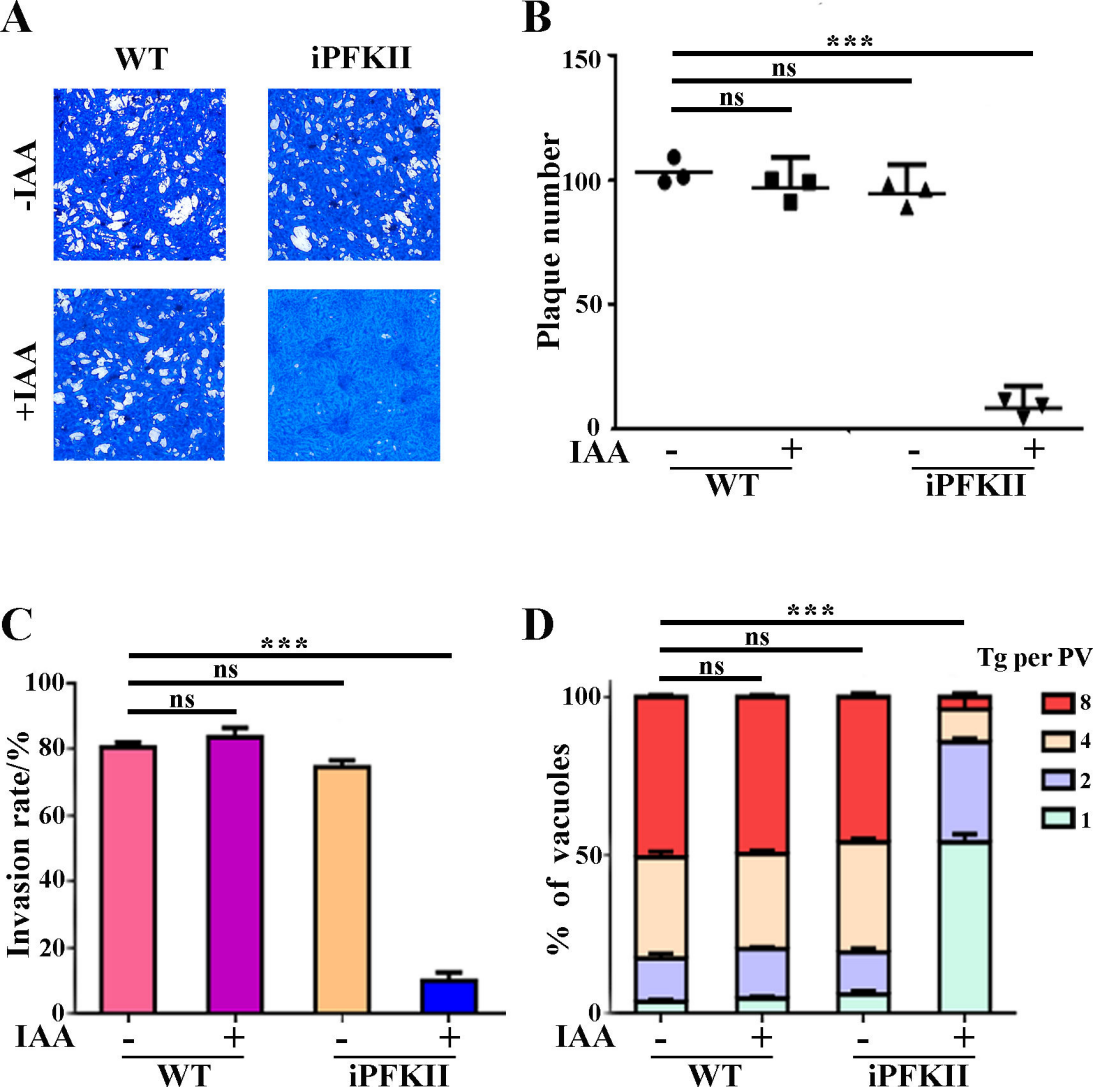
Phenotypic analysis in the conditional knockout strain iPFKII. (**A**) Plaque assays were performed with iPFKII and wild-type RH-TIR1 strains in the presence or absence of IAA. (**B**) Numbers of plaques from A. The experiments were repeated twice independently, each with three replicates. Error bars represent the mean ± SD (*n* = 3). ***P < 0.001. (**C**) Invasion efficiency of Vero cells by the parasites. Error bars represent the mean ± SD (*n* = 3). ***P < 0.001. (**D**) Intracellular replication of parasites in Vero cells. Error bars represent the mean ± SD (*n* = 3). ***P < 0.001.

To investigate the role of PFKII in parasite growth, a plaque assay was conducted using the iPFKII strain in the presence or absence of IAA. The IAA treatment impeded the growth of the iPFKII strain, resulting in a significant decrease in the number of plaques (Fig. 3A and B). Invasion and intracellular replication assays demonstrated that the invasion efficiency and proliferation rate of the IAA-treated iPFKII strain were significantly lower than those of the untreated parasites (Fig. 3C and D). These findings indicate that PFKII is crucial for the invasion and growth of *T. gondii*.

### PFKII is an important regulator of lactylation in *T. gondii*

**Fig 4:**
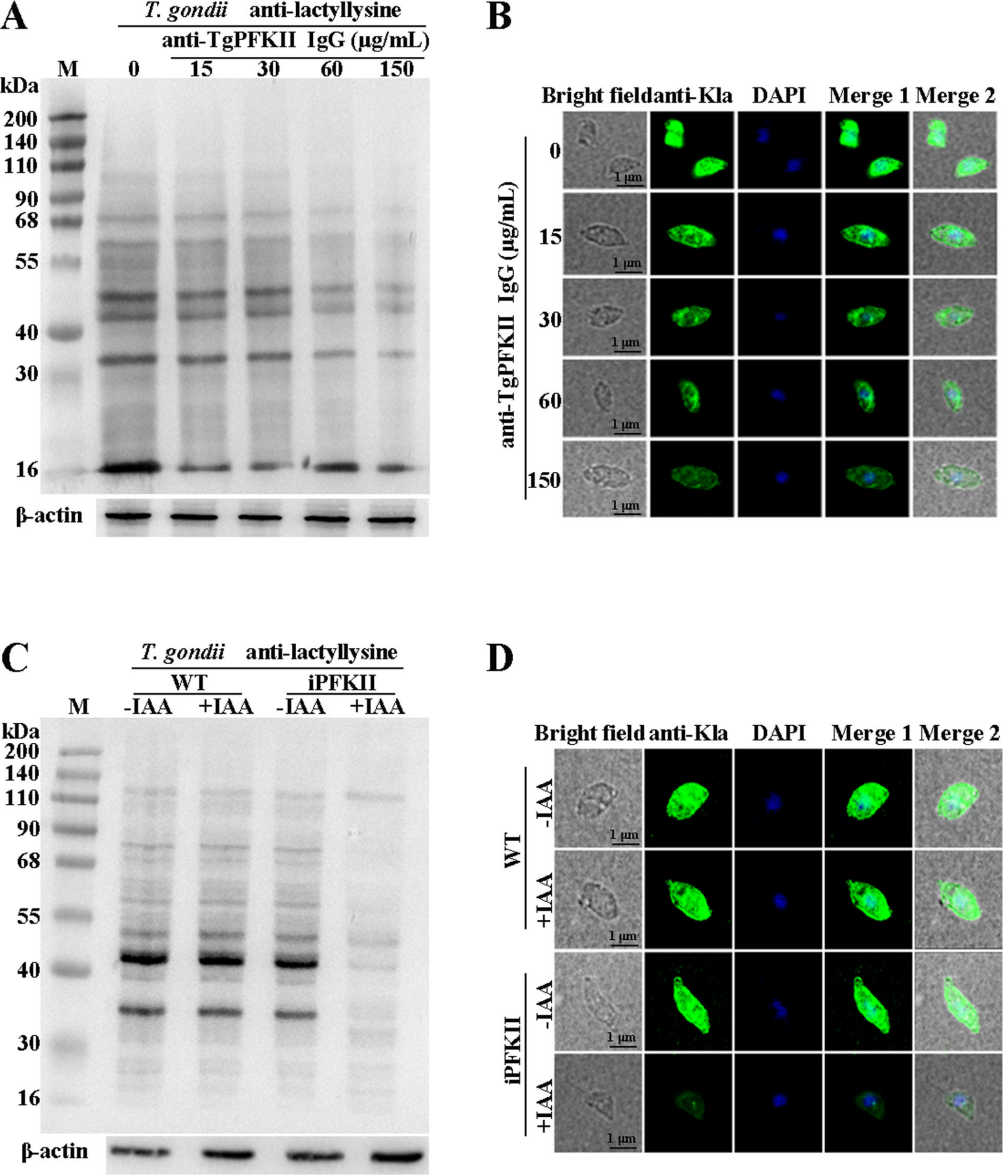
Detection of protein lactylation levels. (**A**) Examination of the effect of different concentrations of PFKII antibodies on *T. gondii* lactylation levels by Western blot analysis. β-Actin was used for normalization. (**B**) Examination of the effect of different concentrations of PFKII antibodies on *T. gondii* lactylation levels (green) by IFA analysis. Scale bar, 1 µm. (**C**) Protein lactylation levels of IAA-treated or non-treated parasites studied by Western blot analysis. β-Actin was used for normalization. (**D**) Lactylation levels of IAA-treated or non-treated parasites (green) studied by IFA analysis. Scale bar, 1 µm.

Our previous studies found that the PFKII antibody can be up-taken by free *T. gondii* tachyzoites and distributed within the cytoplasm (6). Therefore, through the incremental addition of TgPFKII-specific antibodies to *T. gondii* cultures, a concentration-dependent decrease in lactylation levels was observed. This was corroborated by both Western blot and IFA (Fig. 4A and B; Fig. S5C). The lactylation modification in the IAA-treated iPFKII strain was inhibited (Fig. 4C and D; Fig. S5D). In parallel, to assess the potential of lactate in restoring the lactylation modification levels in the knockout strain, lactate was introduced to the TgPFKII knockout *T. gondii*. The results indicated that lactate was capable of partially rescuing the lactylation modification levels (Fig. S6). These data suggest that TgPFKII is an important positive regulator of lactylation in the *T. gondii*.

### TgPFKII is regulated by the AMPK signaling pathway

**Fig 5:**
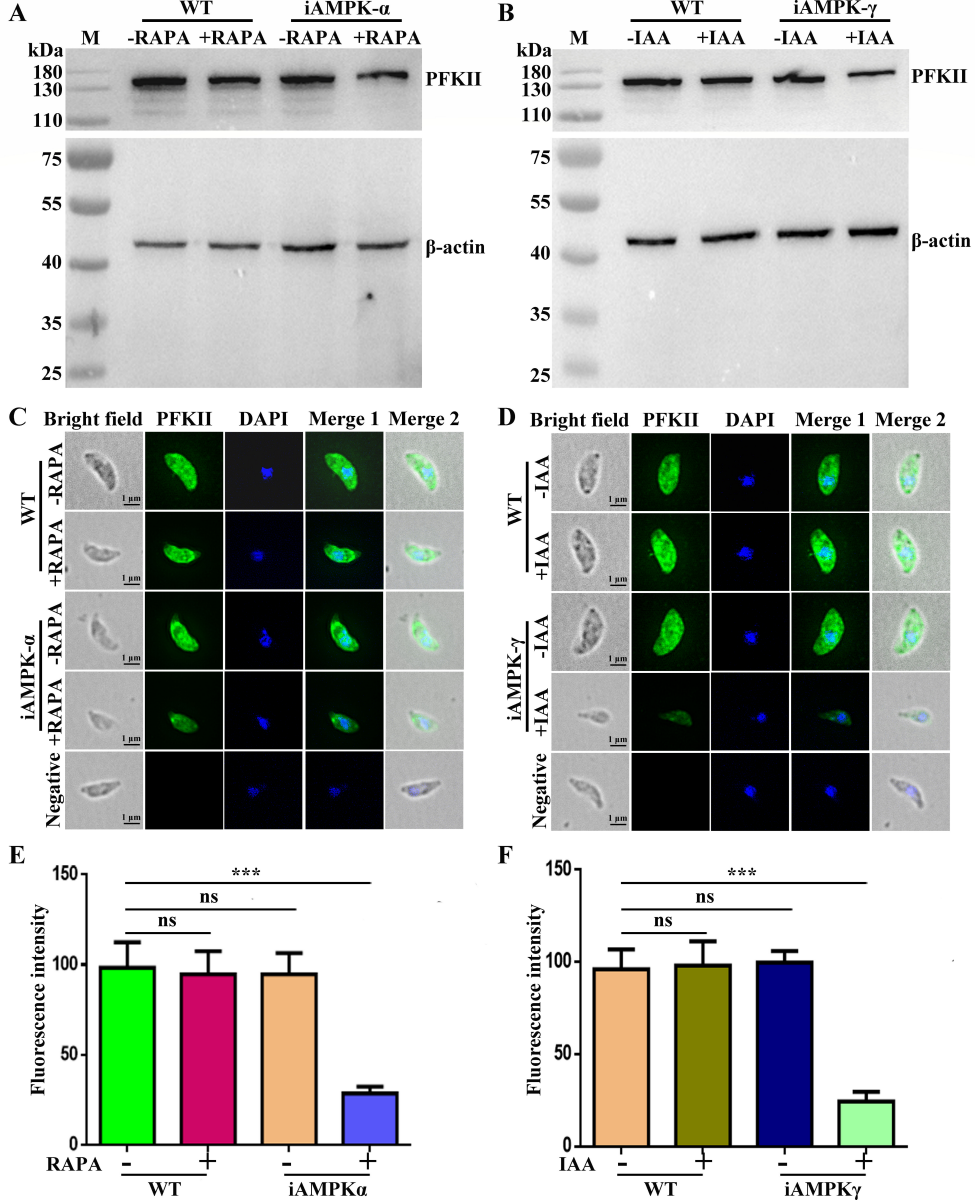
TgPFKII was regulated by the AMPK signaling pathway. (**A–D**) Effect of conditional knockdown of AMPKα and AMPKγ on TgPFKII expression detected by Western blot and IFA analysis. β-Actin was used for normalization. Scale bar, 1 µm. (**E and F**) The immunofluorescence intensities of the conditional knockown strains iAMPKα and iAMPKγ were statistically analyzed separately. Error bars represent the mean ± SD (*n* = 3). ***P < 0.001.

AMPK is known to act as a sensor of cellular energy status and regulates the abundance of glycolysis-related enzymes in *T. gondii* (29). To investigate the regulation of TgPFKII by the AMPK signaling pathway, we examined TgPFKII expression following conditional knockdown of TgAMPKα and TgAMPKγ. Our results revealed that the expression of TgPFKII was significantly decreased upon deletion of AMPKα and AMPKγ (Fig. 5A through F). Furthermore, experiments with AMPK inhibitors (dorsomorphin and Wu-5) demonstrated a dose-dependent reduction in TgPFKII expression (Fig. S7). These findings indicate that TgPFKII expression is modulated by the AMPK signaling pathway.

### Inhibition of the AMPK signaling pathway reduced protein lactylation in *T. gondii*

**Fig 6:**
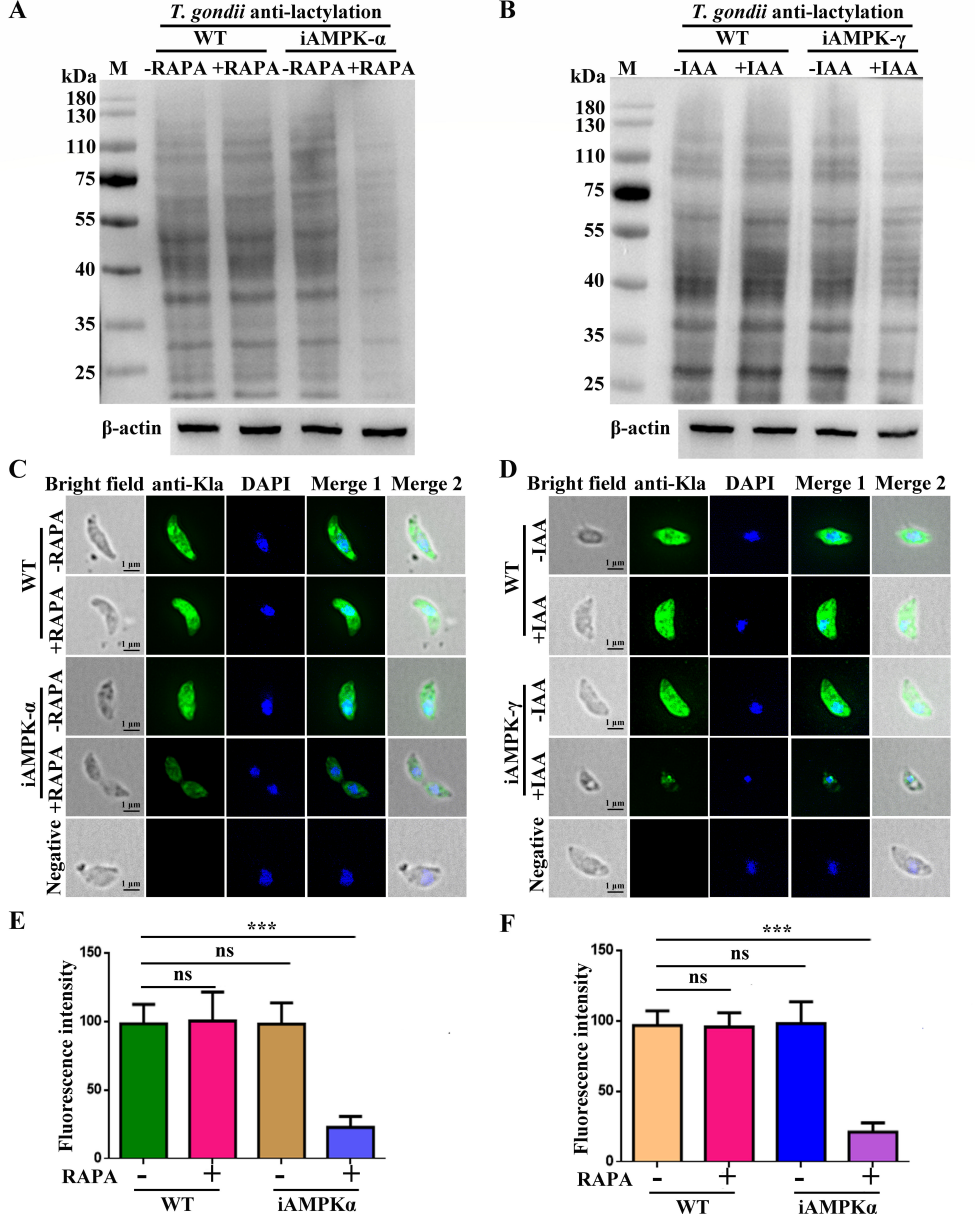
*T. gondii* protein lactylation was regulated by the AMPK signaling pathway. (**A–D**) Lactylation of *T. gondii* iAMPKα and iAMPKγ strains was analyzed by Western blot and IFA analysis. β-Actin was used for normalization. Scale bar, 1 µm. (**E and F**) The lactylation of *T. gondii* was analyzed using an anti-lactyllysine primary antibody in IFA. Fluorescence signal intensity (green) decreased with conditional knockdown of AMPKα and AMPKγ. Error bars represent the mean ± SD (*n* = 3). ***P < 0.001.

To examine the impact of the AMPK signaling pathway on protein lactylation, we used Western blotting and IFAs to detect lactylation levels in conditionally deficient AMPKα and AMPKγ *T. gondii*. The results showed a significant reduction in protein lactylation levels following the conditional knockdown of AMPKα and AMPKγ (Fig. 6A through F). Similarly, experiments with AMPK inhibitors (dorsomorphin and Wu-5) revealed a decrease in lactylation levels (Fig. S8). These findings suggest that protein lactylation in *T. gondii* is regulated by the AMPK signaling pathway.

### The AMPK pathway was involved in the regulation of *T. gondii* invasion and proliferation

**Fig 7:**
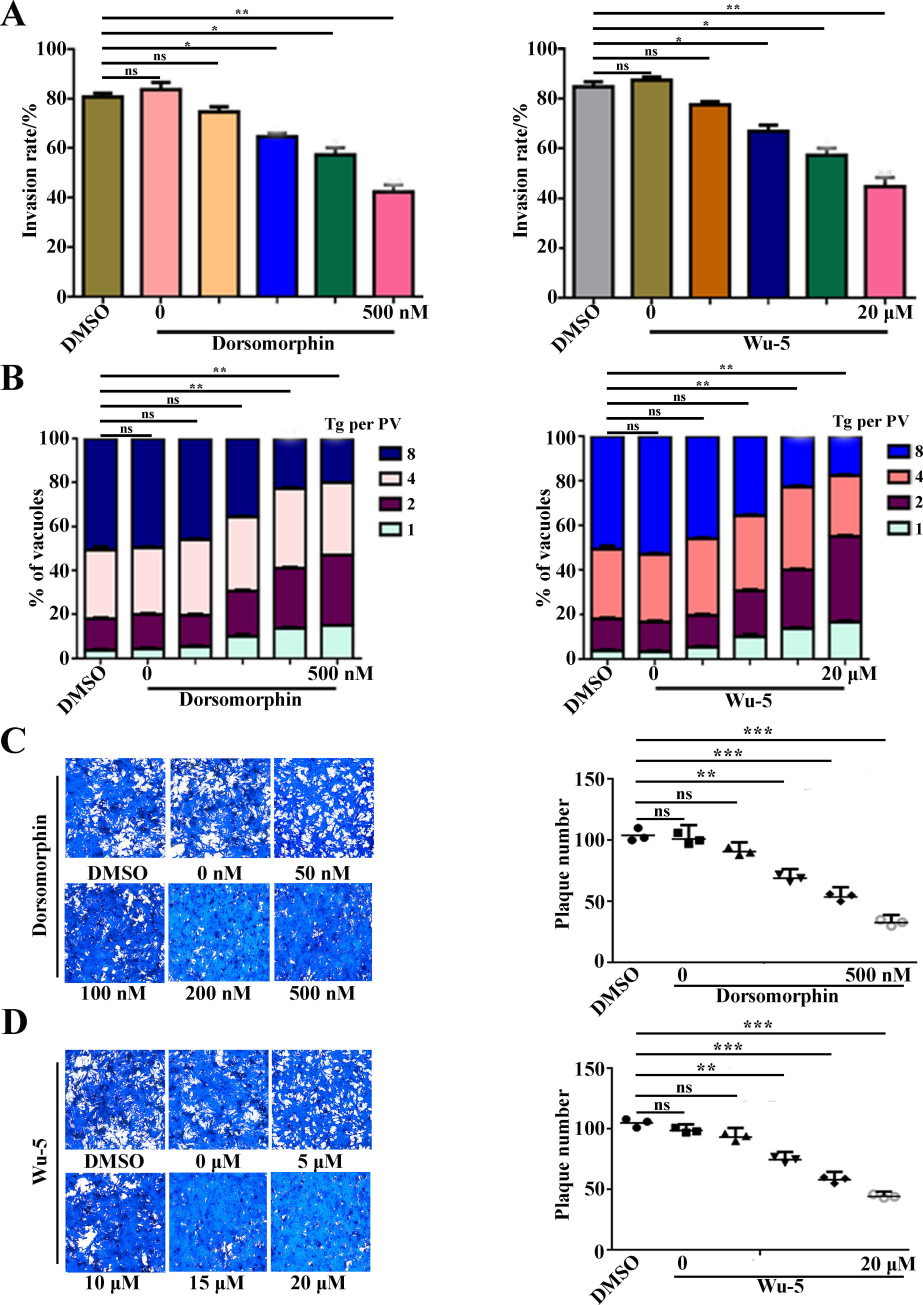
AMPK inhibitors dorsomorphin and Wu-5 inhibit parasite invasion and proliferation. (**A and B**) *T. gondii* invasion and replication rates were reduced by dorsomorphin and Wu-5. Dimethyl sulfoxide (DMSO) was used as a negative control. Error bars represent the mean ± SD (*n* = 3). *P < 0.05. **P < 0.01. (**C and D**) Intracellular growth of *T. gondii* was inhibited by dorsomorphin and Wu-5 in plaque assays. Numbers of plaques from A. The experiments were repeated twice independently, each with three replicates. Error bars represent the mean ± SD (*n* = 3). **P < 0.01. ***P < 0.001.

We treated the parasites with AMPK-specific inhibitors to investigate whether the AMPK signaling pathway can regulate the invasion and replication of *T. gondii*. The invasion and intracellular replication assays showed a significant decrease in parasite invasion efficiency and proliferation with increasing concentrations of AMPK inhibitors (Fig. 7A and B). The plaque assay revealed a significant decrease in the number of plaques with increasing inhibitor concentrations (Fig. 7C and D). These data suggest that the AMPK is involved in the regulation of parasite invasion and growth.

## DISCUSSION

Protein lactylation was initially discovered in human cells (4). Several studies have found that histone lactylation affects a variety of diseases such as tumors, inflammation, infections, and cognitive disorders (4, 5, 8). *Toxoplasma gondii*, belonging to the Apicomplexa, infects nucleated cells of warm-blooded animals and causes toxoplasmosis (4, 32, 33). We first reported that protein lactylation is widely distributed in in *T. gondii* with 1,964 lactylation sites identified on 955 proteins (6). However, the underlying mechanism is still unknown. Based on a previous study (6), we found that the key enzyme TgPFKII in the glycolytic pathway may be involved in the lactylation in *T. gondii*. In this study, we continued our previous work and found that TgPFKII is localized in the cytoplasm (Fig. 1), which is essential for parasite growth (Fig. 2 and 3). Inhibition of the AMPK pathway decreases TgPFKII expression (Fig. 5), reduces protein lactylation levels (Fig. 6), and significantly impairs parasite invasion and proliferation (Fig. 7).

In human and animal cells, knocking down or overexpressing p300 results in reduced or elevated lactylation, respectively (19, 20). Thus, p300 is generally considered the “writer” for lactylation modifications (20–22). In this study, the conditional knockdown of TgPFKII significantly decreased the pan-protein lactylation in *T. gondii* (Fig. 4). This suggests that TgPFKII could also act as a writer of protein lactylation in *T. gondii*. Additionally, AMPK, in mammals, was confirmed to be a highly conserved master regulator of metabolism (23) and a sensor of cellular energy status (24–26). AMPK could phosphorylate more than 100 distinct target proteins on at least 130 sites and modulates almost every branch of cell metabolism and protein function (24, 28). Recently, TgAMPK was identified and characterized as an essential enzyme for the development of *T. gondii* (29–31). The deletion of AMPKγ subunit inhibited protein phosphorylation and functional impairment of an abundance of glycolysis-related enzymes (29). In this study, we utilized the iAMPKα and iAMPKγ strains to explore the effects of the AMPK signaling pathway on TgPFKII expression and *T. gondii* protein lactylation levels. Our findings demonstrated that the conditional knockdown of AMPKα and AMPKγ (29, 31) led to a significant reduction in TgPFKII expression (Fig. 5) and decreased protein lactylation levels (Fig. 6). Consistent results were observed when *T. gondii* RH-TIR1 tachyzoites were treated with the two AMPK pathway inhibitors (dorsomorphin and Wu-5) (Fig. S7 and S8). These findings suggest that AMPK regulates protein lactylation by modulating TgPFKII expression.

The phosphorylation of the TgAMPK subunit impacted many biological processes of *T. gondii*, including invasion, growth, and replication (29–31). However, the mechanism of this has not been clearly understood. In this study, treatment of *T. gondii* RH-TIR1 tachyzoites with AMPK inhibitors resulted in the inhibition of lactylation modifications, as well as a significant reduction in invasion and intracellular proliferative capacity (Fig. 7; Fig. S8). Knockdown of AMPK has been found to reduce *T. gondii* glycolytic flux and the abundance and phosphorylation of related enzymes, affecting the energy metabolic process in *T. gondii* (29). PFKII, as a key metabolic enzyme in the *T. gondii* glycolytic pathway, is regulated by the AMPK signaling. Proteomics analysis showed that *T. gondii* lactylation modification was closely related to the glycolytic pathway and positively correlated with lactate content (6). Another study in the brain found that adding lactic acid to neurons resulted in neuronal excitation along with increased levels of lactation (34). In this study, our findings indicate that the introduction of exogenous lactate to *T. gondii* with TgPFKII knockout partially mitigated protein emulsification levels (Fig. S6). This observation implies that the regulatory mechanisms of TgAMPK in biological processes may encompass the modulation of protein lactylation. However, it is unclear when, how, and why this cascade promotes the *T. gondii* growth and protein lactylation, which needs to be analyzed in further studies.

In mammals, AMPK has been reported to negatively regulate ATP-consuming biosynthetic processes, including gluconeogenesis, lipid and protein synthesis (25, 26, 35). It was shown that AMPK is not essential for the growth and development of mammalian eukaryotic cells under *in vitro* culture conditions (36, 37). In contrast, the protozoan parasite *T. gondii* relies mainly on the glycolytic pathway to produce ATP, and phosphorylation of the AMPK subunit promotes catabolism and ATP biosynthesis in the parasite (29). AMPK has been found to be essential for the survival of *T. gondii*, and deletions of both AMPKγ and AMPKα result in severe growth defects in the parasite (29). This highlights the differences in energy metabolism between *T. gondii* and humans and animals, suggesting that AMPK may execute distinct functions in *T. gondii* and other organisms (29).

In conclusion, our results suggest that conditional knockdown of TgAMPKα and TgAMPKγ results in reduced TgPFKII expression, which ultimately reduced levels of protein lactylation in *T. gondii*. Additionally, AMPK-specific inhibitors significantly impaired parasite invasion and proliferation. This study provides a foundation for understanding the regulatory mechanisms of protein lactylation in *T. gondii*.

## MATERIALS AND METHODS

### Cells and parasites

Vero cells were cultivated in Dulbecco’s Modified Eagle Medium/high glucose medium (Servicebio, Wuhan, China) containing 8% fetal bovine serum at 37°C and 5% CO_2_. The conditional knockout strain of T. gondii used in this study was produced from the RH-TIR1 strain (17, 29).

### Generation of recombinant TgPFKII

The gene encoding TgPFKII (gene ID, *TGGT1_226960*) was obtained from *T. gondii* RH tachyzoites using gene-specific primers by PCR (Table S1). The amplicon was cloned into pET-28a and pGEX-4T-1 vectors (Invitrogen, Carlsbad, CA, USA), respectively. Both recombinant plasmids were expressed in *Escherichia coli* BL21 (DE3) cells (TransGen Biotech, Beijing, China). Soluble recombinant glutathione transferase GST-PFKII and His-PFKII proteins were purified by affinity chromatography as described (Fig. S1) (15).

### Generation of specific antibodies against TgPFKII

Three female SD rats were immunized with purified His-tagged recombinant TgPFKII protein (100 μg/rat) in Freund’s adjuvant (Sigma-Aldrich, St. Louis, MO, USA) by subcutaneous injection for four times to generate specific polyclonal antibodies against TgPFKII (16).

### Expression and immunofluorescence analysis of PFKII in *T. gondii*

To investigate the expression of native TgPFKII, 3 × 10^6^ purified RH-TIR1 tachyzoites were lysed. The lysate was dissolved in 5 × SDS loading buffer (Beyotime, Shanghai, China), electrophoresed on a 10% SDS-PAGE gel, and transferred to a polyvinylidene fluoride (PVDF) membrane (Millipore, Boston, MA, USA). The membranes were blocked with 10 mL phosphate buffered saline (PBS) containing 5% skim milk for 1 h at 37°C, and then incubated in phosphate buffered saline with Tween 20 (PBST) containing rat anti-PFKII specific antibody (1:800) for 13 h at 4°C. After three washes with PBST, the membranes were incubated with alkaline phosphatase (AP)-conjugated goat anti-rat IgG (1:1,000; EASYBIO, Beijing, China) at 37°C for 1 h. The membranes were then washed three times with PBST and developed for 2 min using a BCIP/NBT AP chromogenic kit (Beyotime). Finally, the membranes were scanned with an imaging scanner.

For the IFA, purified RH-TIR1 tachyzoites (1 × 10^6^) were fixed in 4% paraformaldehyde (PFA, Beyotime) and permeabilized on slides with 0.25% Triton X-100 (Beyotime). Parasites were blocked with 1 mL PBS containing 5% skim milk for 45 min at 37°C and incubated for 13 h with a rat anti-PFKII antibody at 4°C (1:100). The slides were then washed four times with PBS and incubated with an Alexa Fluor 488 conjugated goat anti-rat IgG (1:1,000, ThermoFisher Scientific, Waltham, MA, USA) for 45 min at 37℃. After four washes with PBS, the slides were stained with 4′,6-diamidino-2-phenylindole (ThermoFisher Scientific) for 20 min at 25℃ and washed six times with PBS. Samples were examined using a confocal laser scanning microscope (Leica SP8, Wetzlar, Germany) (15, 16).

### Conditional knockdown of TgPFKII using AID fusion approach

The PFKII knockdown strain was named iPFKII. The primers and plasmids used to generate iPFKII in this study are shown in Table S1 and Fig. S2 and S3, respectively. The locus-specific CRISPR construct pSAG1-Cas9-U6-sgPFKII was generated by replacing the UPRT-targeting gRNA in pSAG1-Cas9-U6-sgUPRT with gene-specific gRNAs by Q5 mutagenesis thermal cycling. The PFKII-mAID-Ty-DHFR amplicon was generated from the pmAID-Ty-DHFR plasmid by special primers (Table S1).

A total of 10 μg of the PFKII-mAID-DHFR amplicon was transfected into 1 × 10^7^ RH-TIR1 tachyzoites along with 50 μg of the pSAG1-Cas9-U6-sgPFKII plasmid and selected with pyrimethamine (Sigma) for 7 days. The monoclonal strains were screened by limiting-dilution method, and the monoclonal strains were verified using PCR with specific primers (Table S1), IFA, and Western blot analysis. PFKII degradation in the iPFKII strain was induced by adding IAA (Sigma) to culture medium to a final concentration of 500 μM and incubating for 4 h.

### Phenotypic characterization of iPFKII parasites

Overall growth of the iPFKII strain was analyzed by standard plaque assays as previously described (38). Briefly, Vero cells were divided into four groups, two of which were infected with 50 iPFKII tachyzoites (divided into IAA-treated and untreated groups) and the other two with 50 wild-type RH-TIR1 tachyzoites (divided into IAA-treated and untreated groups). After 7 days, cells were stained with 1 μM crystal violet (Beyotime) for 20 min and images were acquired.

The invasion efficiency of the wild-type RH TIR1 and iPFKII parasites was compared in red/green assays as previously described (38). Briefly, the parasites invaded into six-well plates of Vero cells for 2 h. Parasites that did not invade the cells were washed away with PBS and fixed with 4% PFA for 15 min. Rat anti-SAG2 and a rabbit anti-ROP9 were used to stain extracellular or intracellular parasites. Finally, 10 fields of view were selected for observation under a fluorescence microscope, and the invasion rate of *T. gondii* was calculated.

The proliferation of iPFKII parasites and wild-type RH-TIR1 parasites was compared by the intracellular replication assays as described previously (38). Wild-type RH-TIR1 parasites (both IAA-treated and non-treated groups) and iPFKII parasites (also IAA-treated and non-treated groups) were allowed to invade Vero cells for 4 hours. Uninvaded parasites were removed by washing with PBS. After 24 h of incubation, the number of vacuoles containing one, two, four, and eight parasites were counted by microscopy as described above (18), and 100 vacuoles were counted.

### Detection of protein lactylation in wild-type RH-TIR1 and iPFKII *T. gondii* strains

To test the effect of TgPFKII-specific antibody on protein lactylation, concentrations of 0, 15, 30, 60, and 150 μg/mL of a TgPFKII-specific antibody were added to the Vero cells with parasites and incubated for 5 h at 37°C as described (6). The tachyzoites not invaded were removed by washing with the culture medium. Tachyzoites were collected after 48 h cultivation by centrifugation and lysed by sonication.

To test the lactylation in the iPFKII tachyzoites, RH-TIR1 tachyzoites (IAA-treated and non-treated groups) and iPFKII tachyzoites (IAA-treated and non-treated groups) were collected and lysed as described above. Western blot analysis was performed to examine the levels of lactylation in antibody-treated RH-TIR1 tachyzoites and iPFKII tachyzoites (IAA-treated and untreated groups). An anti-lactyllysine antibody (1:500, catalog no. PTM-1401, PTM BIO, Hangzhou, China) was used as the primary antibody. Horseradish peroxidase (HRP)-conjugated goat anti-rabbit IgG (1:1,000, EASYBIO) was used as the secondary antibody and an anti-β-actin antibody was used for normalization of protein quantitative.

To visualize the lactylated proteins in the RH-TIR1 wild-type and iPFKII T. gondii strains, tachyzoites of RH-TIR1 (incubated with different concentrations of TgPFKII antibody), RH-TIR1 (IAA-treated and untreated groups), and iPFKII (IAA-treated and untreated groups) were respectively fixed in 4% PFA (Beyotime) and permeabilized on slides using 0.25% Triton X-100 (Beyotime), and incubated with an anti-lactyllysine antibody (1:200). Alexa Fluor 488-conjugated goat anti-rabbit IgG was used as the secondary antibody (1:1,000, ThermoFisher Scientific). The fluorescence signals were recorded in the same way as described above.

To determine if lactate could restore protein lactylation in the TgPFKII knockout strain, lactate was added and lactylation levels were assessed via Western blot. The primary antibody used was anti-lactyllysine (1:500, PTM-1401, PTM BIO), with HRP-conjugated goat anti-rabbit IgG (1:1,000, EASYBIO) as the secondary antibody, and anti-β-actin for protein normalization.

### Analysis of TgPFKII expression after conditional deletion of AMPKα and AMPKγ

To investigate the effect of the AMPK pathway on TgPFKII activity, we collected conditional knockdown AMPKα and AMPKγ in *T. gondii* and lysed by sonication (29, 31). TgPFKII expression in the two parasite strains was assessed by Western blot and IFAs. For Western blot assays, an anti-TgPFKII antibody was used as the primary antibody at a dilution of 1:800, and HRP-conjugated goat anti-rat IgG (1:1,000, EASYBIO) was used as the secondary antibody. In IFA, the anti-TgPFKII antibody (1:100) served as the primary antibody, and Alexa Fluor 488-conjugated goat anti-rat IgG (1:1,000, ThermoFisher Scientific) was used as the secondary antibody. β-Actin was used for normalization in Western blot assays.

In a separate experiment, the AMPK inhibitors dorsomorphin (MCE, Monmouth Junction, NJ, USA) and Wu-5 (MCE) were prepared at five different concentrations (dorsomorphin: 0, 50, 100, 200, and 500 nM; Wu-5: 0, 5, 10, 15, and 20 μM) (39, 40). Purified RH-TIR1 parasites were incubated with the inhibitors at 37°C for 4 h. Following treatment, 1 × 10⁴ inhibitor-treated parasites were added to Vero cells and cultivated at 37°C for 72 h. Afterward, the parasites were collected by centrifugation, lysed by sonication, and analyzed for TgPFKII expression using Western blotting and IFA as described previously.

### Effect of conditional knockout of AMPKα and AMPKγ on protein lactylation in *T. gondii*

To evaluate the effect of the AMPK pathway on *T. gondii* lactylation levels, parasites with conditional AMPKα and AMPKγ knockdown were collected and lysed by sonication (29, 31). Western blotting and IFAs were employed to analyze the impact of conditional knockdown of AMPKα and AMPKγ on lactylation in *T. gondii*, as described previously. An anti-lactyllysine antibody was used as the primary antibody in Western blot assays (1:500) and IFAs (1:200). HRP-conjugated goat anti-rabbit IgG (1:1,000, EASYBIO) and Alexa Fluor 488-conjugated goat anti-rabbit IgG (1:1,000, ThermoFisher Scientific) were used as secondary antibodies for Western blotting and IFA, respectively. β-Actin was used for normalization in Western blot assays.

In a separate experiment, the AMPK inhibitors, dorsomorphin and Wu-5, were prepared at four different concentrations (dorsomorphin: 0, 100, 200, and 500 nM; Wu-5: 0, 10, 15, and 20 μM) and added to purified RH-TIR1 parasites. The parasites were incubated at 37°C for 4 h. Following inhibitor treatment, 1 × 10⁴ treated parasites were added to Vero cells and cultivated at 37°C for 72 h. After incubation, the parasites were collected by centrifugation, lysed by sonication, and analyzed for the effect of AMPK inhibitors on *T. gondii* lactylation using Western blotting and IFA, as previously described.

### Phenotypic characterization of *T. gondii* after inhibition of the AMPK pathway

Different concentrations of AMPK inhibitors (dorsomorphin: 0, 50, 100, 200, and 500 nM; Wu-5: 0, 5, 10, 15, and 20 μM) were added to the purified RH-TIR1 parasites and incubated at 37°C for 4 h. The same concentration (vol/vol) of dimethyl sulfoxide (Beyotime) was used as a no treatment control. Inhibitor-treated parasites (1 × 10^4^) were added to Vero cells for invasion in six-well plates for 2 h. Standard plaque assays, red/green assays, and intracellular replication assays were used to assess the overall growth, invasion rates, and proliferation of parasites, as previously described.

### Statistical analysis

All data were analyzed using the statistical software GraphPad Prism 5.0 (GraphPad Software, Inc., USA). Two groups of the data were compared using *t*-test, and multiple groups of data were compared using one-way analysis of variance. The mean and standard deviation were determined using three biological replicates. Statistical thresholds of *P* < 0.05, *P* < 0.01, and *P* < 0.001 were considered significant.

## Data Availability

All data were generated or analyzed during this study are included in this published article.

## SUPPLEMENTAL MATERIAL

Supplemental figures spectrum.02044-24-s0001.docx Fig. S1 to S9.

Table S1 spectrum.02044-24-s0002.docx The primers used in this study.

ASM does not own the copyrights to Supplemental Material that may be linked to, or accessed through, an article. The authors have granted ASM a non-exclusive, world-wide license to publish the Supplemental Material files. Please contact the corresponding author directly for reuse.
