# Supplementary material for: The TgAMPK-TgPFKII axis essentially regulates protein lactylation in the zoonotic parasite Toxoplasma gondii
Source: Microbiol Spectr. 2025 Feb 7;13(3):e02044-24. doi: 10.1128/spectrum.02044-24 (PMC11878075; doi:10.1128/spectrum.02044-24)
Supplement: Supplemental figures — Fig. S1 to S9. [file spectrum.02044-24-s0001.docx]

**Supplementary Figures:**


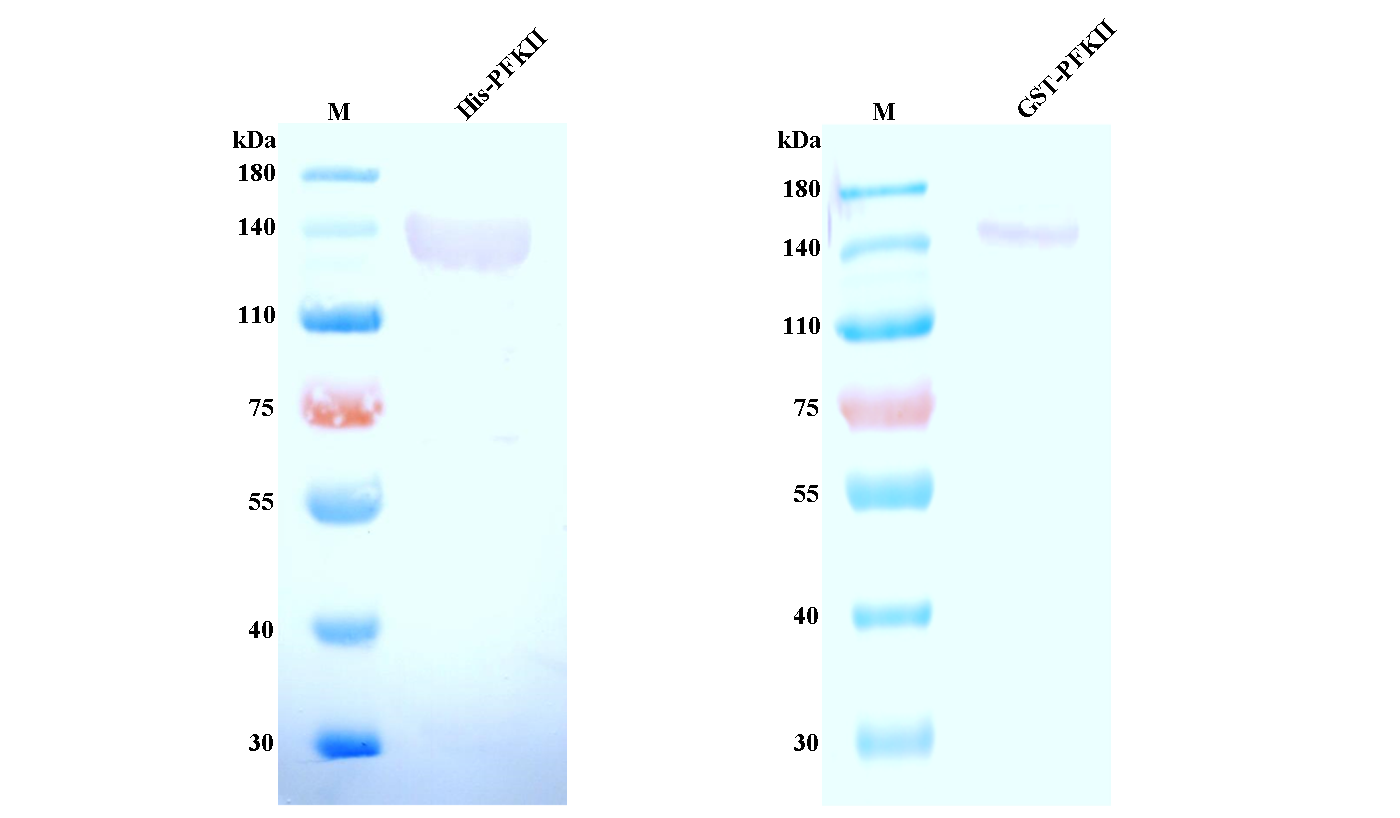


**FIG S1.** His-tagged and GST-tagged PFKII recombinant proteins were detected by Western blots with tag-specific antibodies.


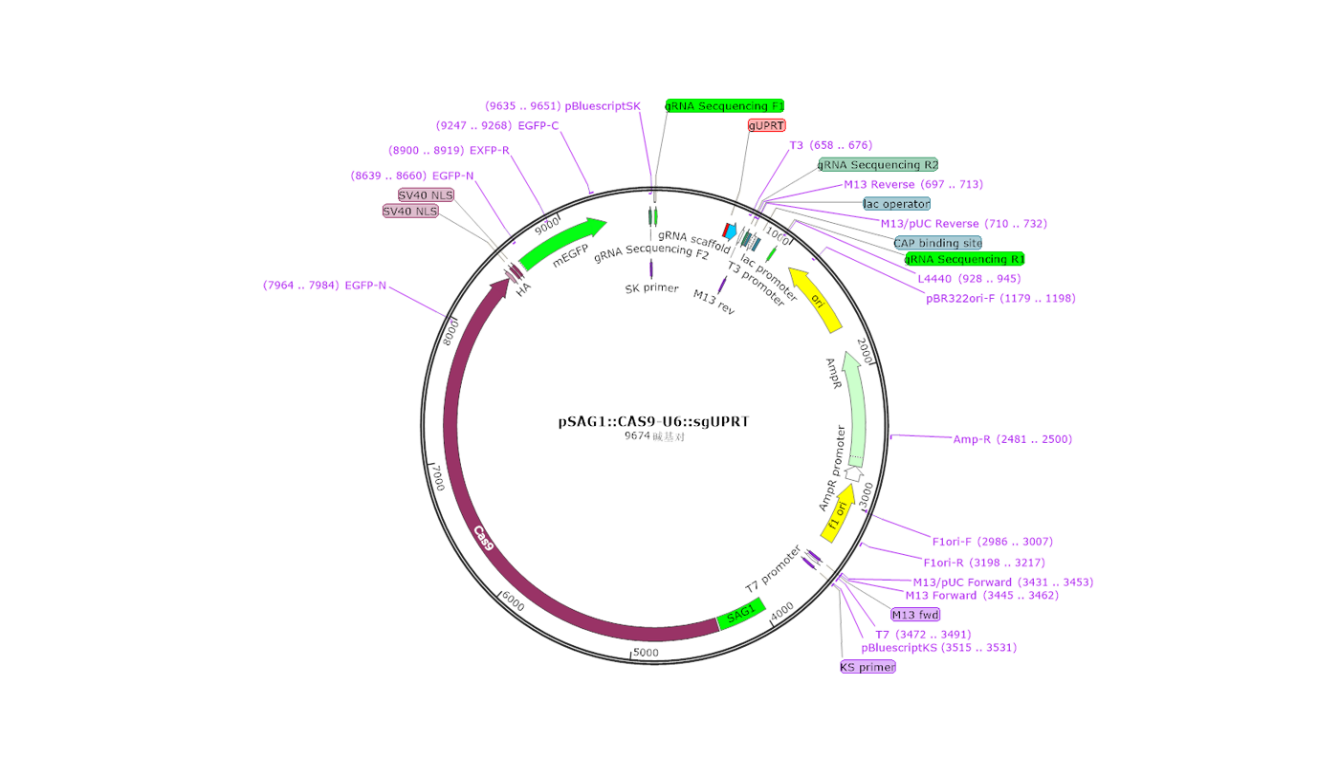


**FIG S2.** Plasmid pSAG1-Cas9-U6-sgUPRT used in the AID conditional knockout system.


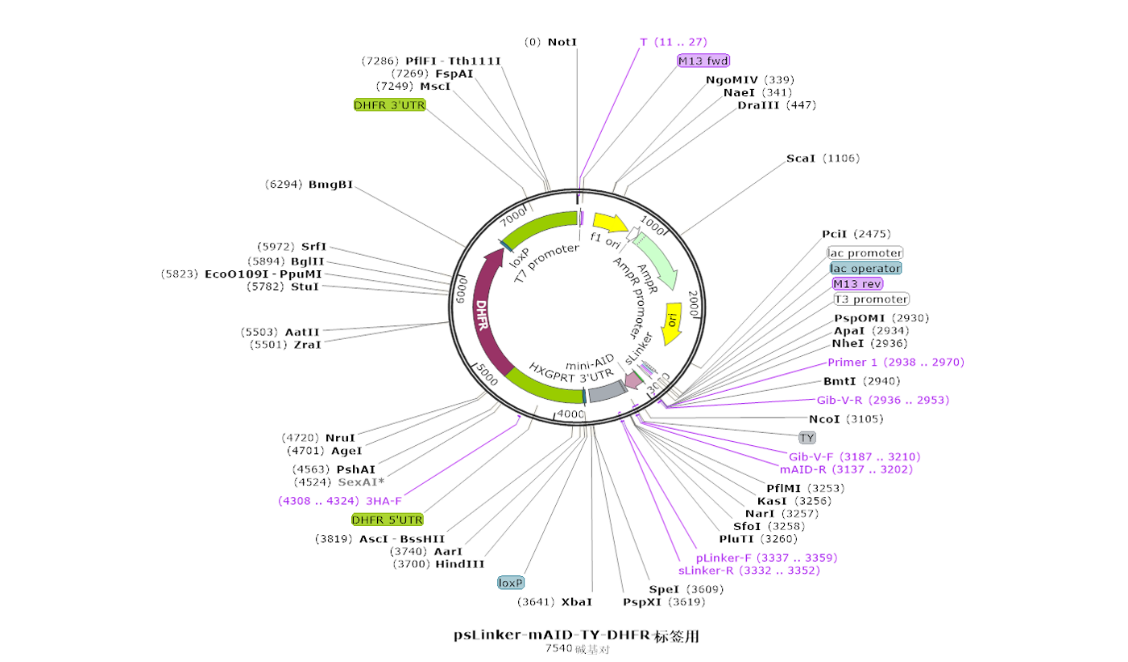


**FIG S3.** Plasmid pmAID-TY-DHFR used in the AID conditional knockout system.


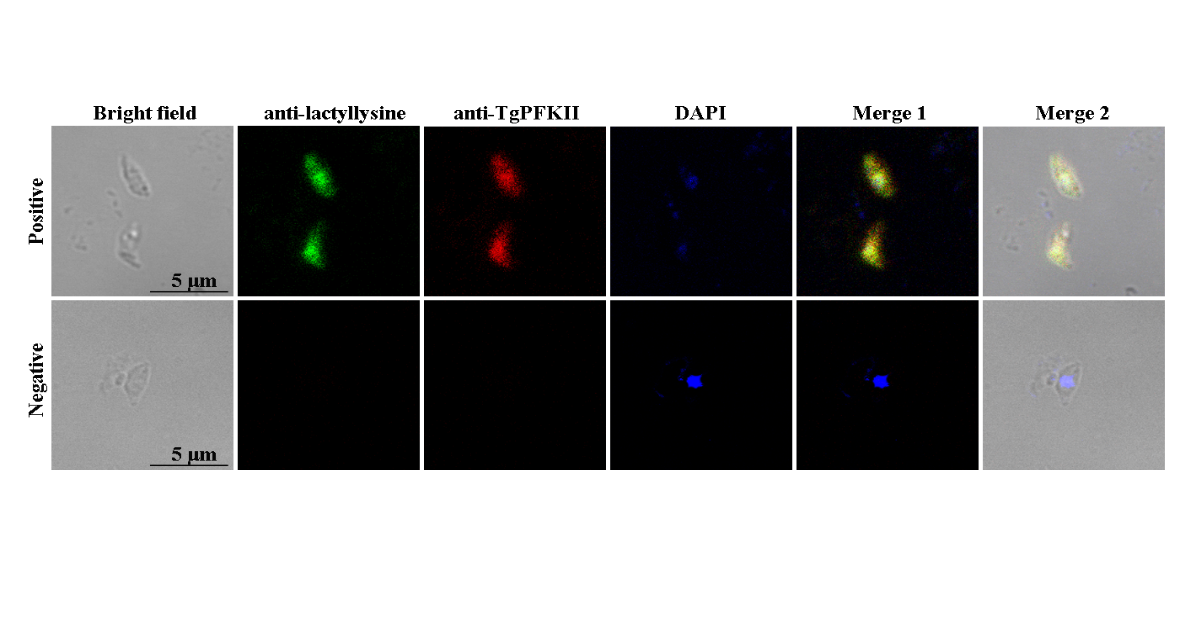


**FIG S4.** The fluorescence signal of lactylation (green) overlaps with that of the TgPFKII (red) in *T. gondii* RH-TIR1 tachyzoites. A serum of a healthy rat was used as the negative control. Scale bar, 5 µm.


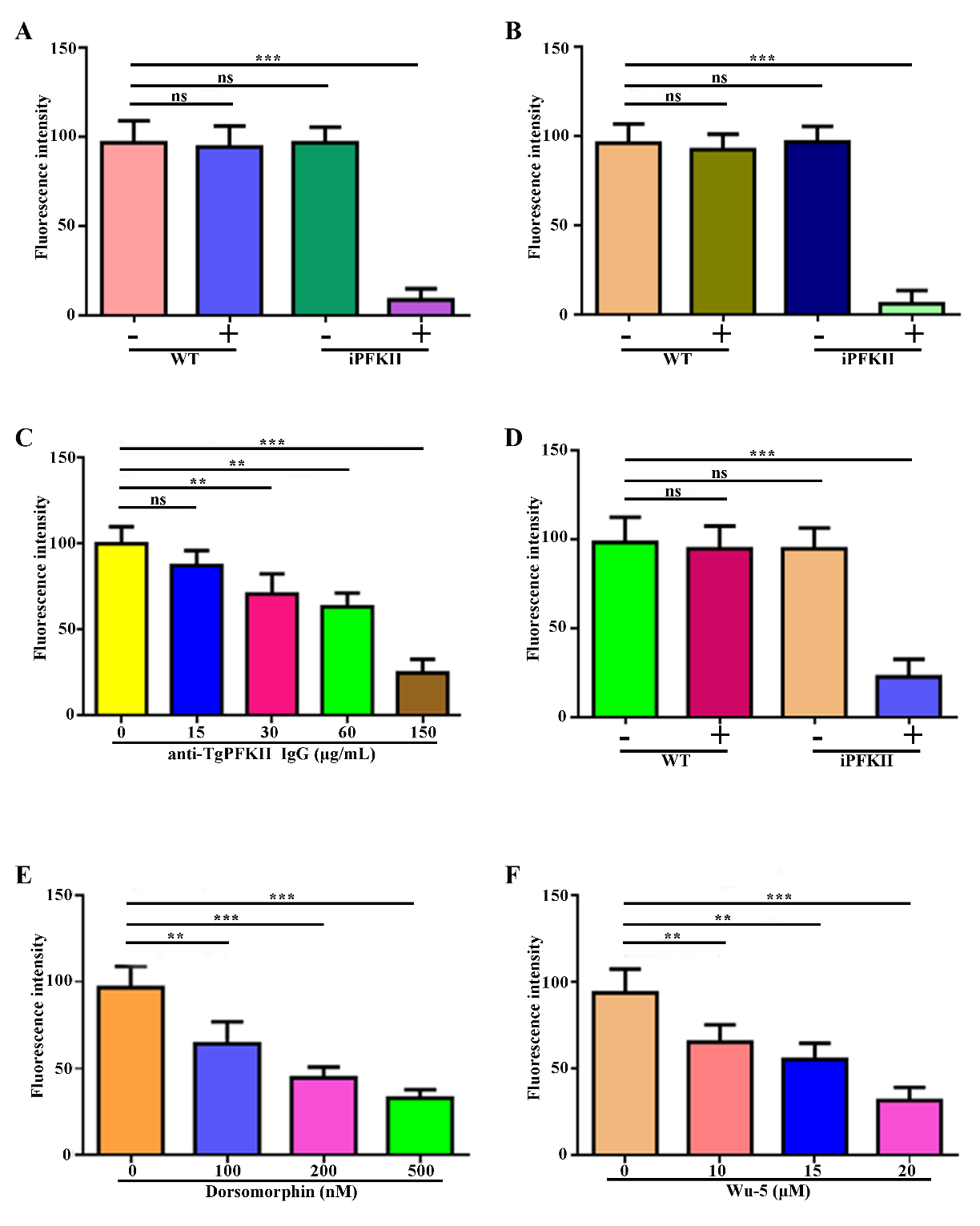


**FIG S5.** Quantification of immunofluorescence intensity. (A) Immunofluorescence intensities of Figure 2D were statistically analyzed. Error bars represent mean ± SD (n = 3). ****p* < 0.001. (B) Immunofluorescence intensities of Figure 2E were statistically analyzed. Error bars represent mean ± SD (n = 3). ****p* < 0.001. (C) Immunofluorescence intensities of Figure 4C were statistically analyzed. Error bars represent mean ± SD (n = 3). ****p* < 0.001. ***p* < 0.01. (D) Immunofluorescence intensities of Figure 4D were statistically analyzed. Error bars represent mean ± SD (n = 3). ****p* < 0.001. (E) Immunofluorescence intensities of Figure 6C were statistically analyzed. Error bars represent mean ± SD (n = 3). ****p* < 0.001. ***p* < 0.01. (F) Immunofluorescence intensities of Figure 6D were statistically analyzed. Error bars represent mean ± SD (n = 3). ****p* < 0.001. ***p* < 0.01.


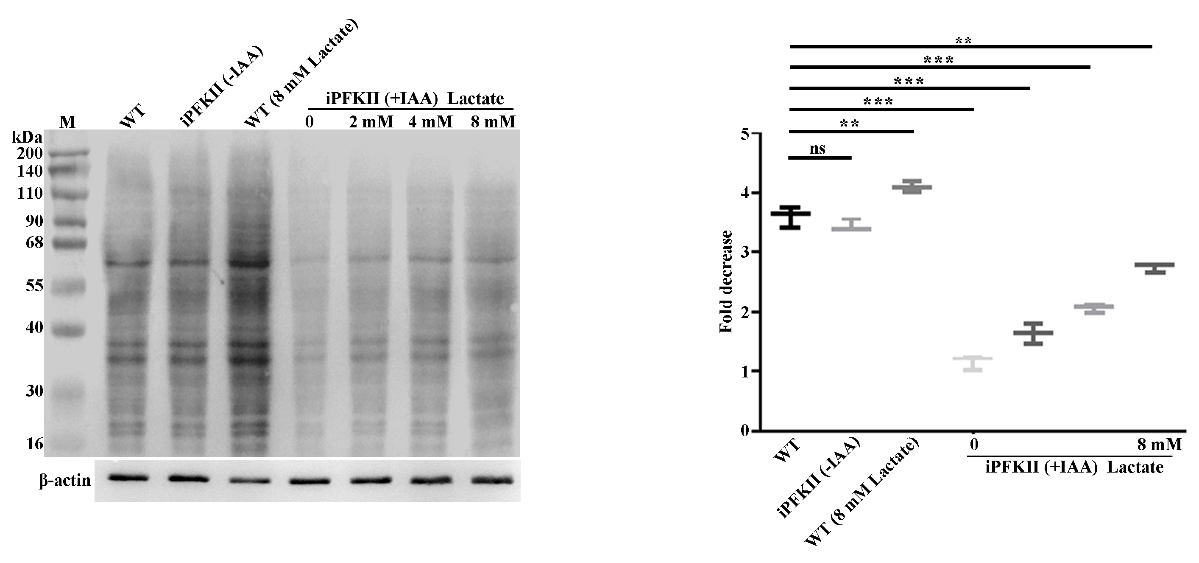


**FIG S6.** Addition of exogenous lactate to TgPFKII knock-down parasites partially alleviated protein lactylation levels. β-actin was used for normalization. Error bars represent mean ± SD (n = 3). ****p* < 0.001. ***p* < 0.01.


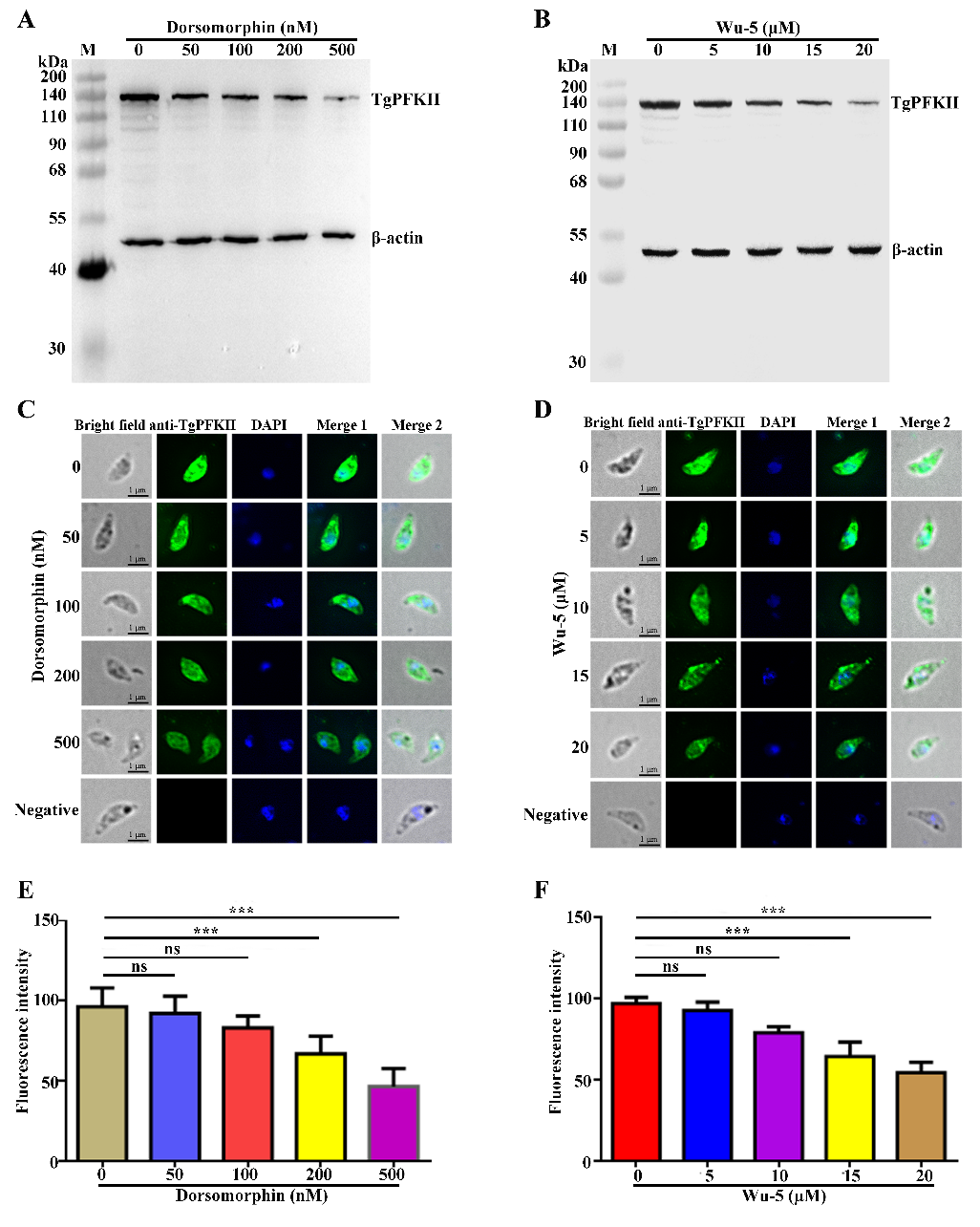


**FIG S7.** TgPFKII was regulated by the AMPK signaling pathway. (A-D) The effect of inhibitors Dorsomorphin and Wu-5 on TgPFKII expression was detected by Western blot and IFA analysis. β-actin was used for normalization. Scale bar, 1 µm. (E-F) The immunofluorescence intensity was statistically analyzed for the different concentration of drug treated groups, respectively. Error bars represent the mean ± SD (n = 3). ****p* < 0.001.


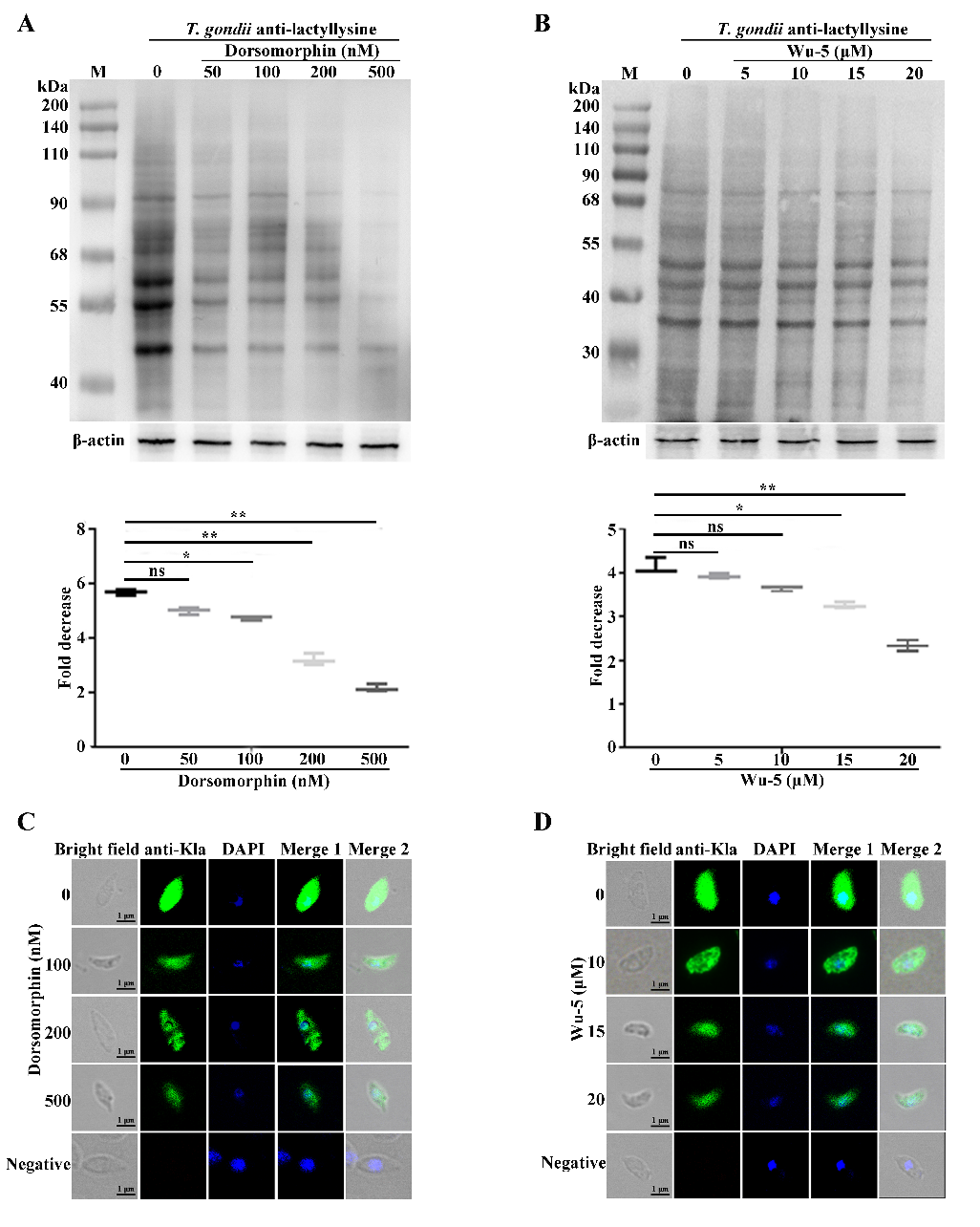


**FIG S8.** *T. gondii* protein lactylation was regulated by the AMPK signaling pathway. (A-B) *T. gondii* lactylation was analyzed by Western blotting after treatment with different concentrations of Dorsomorphin and Wu-5. β-actin was used for normalization. Error bars represent the mean ± SD (n = 3). **p* < 0.05. ***p* < 0.01. (C-D) The lactylation of *T. gondii* was analyzed using an anti-lactyllysine primary antibody in IFA. The intensity of the fluorescence signal (green) decreased with increased concentrations of the inhibitors. Scale bar, 1 µm.


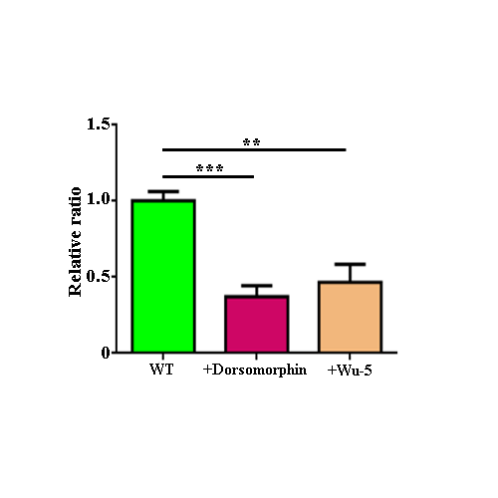


**FIG S9.** Dorsomorphin and Wu-5 inhibited the expression of AMPK as verified by quantitative PCR. ***p* < 0.01. ****p* < 0.001.
