# Supplementary material for: The TgAMPK-TgPFKII axis essentially regulates protein lactylation in the zoonotic parasite Toxoplasma gondii
Source: Microbiol Spectr. 2025 Feb 7;13(3):e02044-24. doi: 10.1128/spectrum.02044-24 (PMC11878075; doi:10.1128/spectrum.02044-24)
Supplement: Table S1 — The primers used in this study. [file spectrum.02044-24-s0002.docx]

**Table S1.** The primers used in this study.

| **Primers** | **sequence (5’-3’)** |
| --- | --- |
| PFKII-primer F | ATGACTTTCTTGTCCTTCTTTAAGTGCG |
| PFKII-primer R | TTGGTCTCTGCATGCAGCC |
| PFKII-sgRNA F | GAGTTTGGAATTCTCTGTCGGTTTTAGAGCTAGAAATAGC |
| PFKII-sgRNA R | AACTTGACATCCCCATTTAC |
| 5‘-PFKII-AID | GAAAAGCGAAGCGTGCGGCGCGGCTGCATGCAGAGACCAAGCTAGCAAGGGCTCGGGC |
| 3‘-PFKII-AID | CCCAGAGCCTCTGGTGTCGTCTCAGTTGTGCTCTGACTGGATAGGGCGAATTGGAGCTCC |
| AID-PFKII-PCR1 F | CTGGATTTCCTTGTCGCTCT |
| AID-PFKII-PCR1 R | GTTGTGCTCTGACTGGC |
| AID-PFKII-PCR2 F | CTGGATTTCCTTGTCGCTCT |
| AID-PFKII-PCR2 R | AAATCAATCTTTCGCAGGT |
